# The Cellular Response to DNA Damage: From DNA Repair to Polyploidy and Beyond

**DOI:** 10.3390/ijms24076852

**Published:** 2023-04-06

**Authors:** Razmik Mirzayans

**Affiliations:** Department of Oncology, Cross Cancer Institute, University of Alberta, Edmonton, AB T6G 1Z2, Canada; razmik.mirzayans@ahs.ca

## 1. Special Issue Objectives

A major challenge in treating patients with solid tumors is posed by intratumor heterogeneity, with different sub-populations of cancer cells within the same tumor exhibiting therapy resistance through different biological processes. These include therapy-induced dormancy (durable proliferation arrest through, e.g., polyploidy and senescence), anastasis (a cell recovery phenomenon that rescues cancer cells from the brink of apoptotic and other modes of cell death), and cell fusion. Unfortunately, such responses are often overlooked or misinterpreted as “death” in commonly used preclinical assays, including the in vitro colony-forming assay, multi-well plate cell “viability” or “cytotoxicity” assays, various apoptosis assays (e.g., TUNEL), and tumor growth kinetics in live animals. Although these assays predominantly determine the ability of a given anticancer treatment to convert dangerous (proliferating) cancer cells into potentially even more dangerous (dormant) cancer cells, the results are often assumed to reflect cancer cell demise. It is therefore not surprising that after half a century of extensive research and numerous clinical trials, cancer remains the second-leading cause of death in developed countries [1].

The purpose of this Special Issue, entitled “The Cellular Response to DNA Damage: From DNA Repair to Polyploidy and Beyond”, is to provide a comprehensive update on the growing complexity of molecular and cellular responses to genotoxic stress. Invitations to submit manuscripts were extended to authors who utilize single-cell biology to study the long-term fate of human cells following treatment with ionizing radiation or chemotherapeutic drugs. Three original articles and five reviews were published, most of which focused on the creation and fate of polyploid/senescent giant cancer cells that have emerged as the root causes of therapy resistance and disease recurrence [2].

## 2. Research Articles

Bojko et al. [3] reported a series of studies determining the relationship between doxorubicin-induced senescence, polyploidy, and autophagy (a self-degradative process) in breast cancer cell lines with differing p53 statuses. Experiments were performed under conditions (duration and concentration of drug treatment) that were consistent with how drugs are administered to patients. Breast cancer cells expressing wild-type p53 (MCF7) or mutant p53 (MDA-MB-231) underwent dormancy (senescence/polyploidization) following doxorubicin treatment. Subsequently, dormant giant cells produced proliferating progeny called “escapers” that exhibited nuclear contents comparable to pretreated parental cells. Autophagic influx (a measure of autophagic degradation activity) was shown to be indispensable in breast cancer cell escape from senescence/polyploidy-associated dormancy.

Salmina et al. [4] carried out studies that were designed to explore the consequences of mitotic slippage (the incomplete mitosis that results in a doubled genome in the interphase) in the MDA-MB-231 breast cancer cell line after doxorubicin treatment. Several phenomena were observed, including cellular senescence, polyploidization by mitotic slippage, the activation of meiotic genes, extranuclear sorting of the cut-off circularized telomere ends, and finally, an amoeba-like asexual life-cycle. These complex processes enable the return of the progeny of dormant (polyploid/senescent) cancer cells into the mitotic cycle. The authors provided an in-depth discussion, placing all these complex processes into a mutual context and explaining, in particular, why mitotic slippage improves cell survival.

Polyploidy and atavistic reprogramming (the switch to a unicellular form of life) are two features of cancer that underlie therapy resistance. Anatskaya et al. [5] undertook a comparative phylostratigraphic analysis of ploidy-related genes that were obtained from transcriptomic data of polyploid and diploid human and mouse tissues, and provided evidence suggesting that, in cancer, the atavistic shift goes hand in hand with polyploidy and is driven by epigenetic mechanisms that are favoured by the activation of bivalent genes (exhibiting both active and repressive features, a hallmark of pluripotency) and the deregulation of circadian rhythms. This study, therefore, highlighted the paramount role of polyploidy in the atavistic origin of cancer, which is one of the reasons for the incurability of the metastatic disease.

## 3. Reviews

Fitieh et al. [6] supported this Special Issue by reviewing our current understanding of the role of polycomb group protein BMI1 in epigenetic gene silencing, DNA repair, and genomic stability. The authors concluded that targeting this multifunctional protein via several different mechanisms may provide therapeutic strategies for BMI1-overexpressing cancers.

A comprehensive review on the XPA (xeroderma pigmentosum complementation group A) protein was provided by Pulzová et al. [7], which included the mechanisms of transcriptional regulation of XPA as well as its function and interacting partners both in and outside nucleotide excision repair. In addition, the authors reviewed recent clinical studies suggesting that XPA might be used as a potential prognostic and predictive biomarker for the response to anticancer treatment.

Storozynsky and Hitt [8] reviewed the current state of our understanding of the interplay between ionizing radiation-induced DNA injury and innate immune signaling in the context of cancer radiotherapy. The authors first discussed the mechanisms by which radiation exposure leads to the activation of a cytosolic DNA-sensing pathway mediated by the cyclic GMP-AMP (cGAMP) synthase (cGAS) and stimulator of interferon genes (STING), which initiates the innate immune signaling that facilitates adaptive antitumor immune responses. In addition, they reported on factors that modulate cGAS–STING signaling, deleterious effects associated with cGAS–STING activation, and promising therapeutic candidates currently under study in combination with radiotherapy to bolster immune activation.

Dörnen et al. [9] provided a comprehensive overview of the key role played by the biological phenomenon of cell fusion in wound healing, tissue regeneration, and other physiological processes. The review focused on the mechanisms of cell fusion, factors that promote cell fusion, and the potential of cell fusion-associated polyploidy, aneuploidy, and genomic instability to promote malignant transformation.

My colleague and I [10] reviewed the significance of fusions between different cell types (cancer cells and cancer cells, cancer cells and leukocytes, cancer cells and stem cells, and cancer cells and stromal cells) in tumor progression and therapy resistance.

This review published by our group [10] provided updates on intratumor heterogeneity reflecting not only cell fusion, but also therapy-induced cancer cell dormancy and anastasis, as outlined above (Section 1). We also discussed the growing realization of the danger of relying on short-term assays (e.g., multi-well plate cell “viability”), as well as TUNEL, caspase activation, and other apoptosis assays, for assessment of cancer cell death following therapeutic exposures [10,11].

In our most recent review [12], published in part 2 of this Special Issue [13], we discussed the reasons for continuing failures in cancer therapy, whether misleading/inappropriate preclinical assays are to be blamed for this “failed medicine,” and whether some modern therapies (with or without surgery) cause more harm than benefit in the treatment of patients with solid tumors. We provided a summary of editorials/presentations by accomplished oncologists on a number of important issues that have hampered progress in cancer therapy. The lecture entitled “Preclinical Cancer Target Validation: How Not to be Wrong” [14] by the Nobel Prize Laureate William Kailen highlighted the potential danger of relying on various “down” assays (e.g., immunoblotting and tumor growth delay in live animals) in anticancer research, as well as relying on the widely used Kaplan–Meier plots in the context of molecular biomarkers.

## 4. Conclusions

I trust that this Special Issue has provided sufficient insight for the reader to elaborate or debate on the main conclusion that my colleague and I have reached [12]. Namely, that “there is urgent need for designing preclinical anticancer assays (both cell-based and animal models) and treatment strategies that recapitulate the degree of complexity that exists within an individual solid tumor. Until then, at least in the near future, perhaps efforts of cancer researchers should be primarily directed towards prevention rather than employing the same misleading preclinical assays and wishy-washy interpretations (to quote Kaelin [14]) with “novel” anticancer drugs and catchy names for treatment strategies (e.g., “synthetic lethality”) to expect different outcomes…”.
